# The Role of Autophagy in Chondrocyte Metabolism and Osteoarthritis: A Comprehensive Research Review

**DOI:** 10.1155/2019/5171602

**Published:** 2019-04-14

**Authors:** Pan Luo, Fuqiang Gao, Dongsheng Niu, Xichun Sun, Qiang Song, Chongjun Guo, Yuqi Liang, Wei Sun

**Affiliations:** ^1^Department of Orthopedics, China-Japan Friendship Hospital, China-Japan Friendship Institute of Clinical Medicine, Chinese Academy of Medical Sciences, Peking Union Medical College, Graduate School of Peking Union Medical College, Beijing 100029, China; ^2^Department of Orthopedics, People's Hospital of Ningxia Hui Autonomous Region, Yinchuan, Ningxia Hui Autonomous Region 750001, China

## Abstract

Chondrocytes are the sole cellular constituents of normal cartilage. The degeneration and apoptosis of these cells are considered the main cause of osteoarthritis (OA). Previous studies have suggested that the enhancement of autophagy in chondrocytes can delay the progression of osteoarthritis by affecting intracellular metabolic activity, i.e., by regulating the metabolism of nutrients, which can delay cell aging and death. In this review, we explored the relationship between autophagy and chondrocyte metabolism and provided new ideas for the prevention and treatment of OA.

## 1. Introduction

Mesochondrium, also known as the matrix of cartilage, is produced by the chondrocytes. Its main function is maintaining the normal structure and function of the cartilage, while its reduction affects the survival of chondrocyte and may lead to osteoarthritis (OA). In addition, the destruction of chondrocytes causes the decrease of cartilage matrix and hinders the nutrition and metabolism of articular cartilage.

Autophagy, also known as type II programmed cell death, is a lysosomal degradation pathway essential for cell survival. So far, many studies have suggested that autophagy is an antiapoptotic cell protection mechanism used by multiple tissue cells [1, 2]. The degeneration and apoptosis of chondrocytes have been considered the main reasons for the development of OA, given that autophagy aids the prevention of cell apoptosis by affecting the cell metabolism.

## 2. Process of Autophagy

During cell hypoxia, low energy supply or other stimuli, autophagy protects the organisms from normal or pathological aging by converting damaged organelles and proteins into the cells [3]. There are three types of autophagy—chaperone-mediated autophagy (CMA), macroautophagy, and microautophagy. The term “autophagy” usually indicates macroautophagy unless otherwise specified.

**Figure 1:**
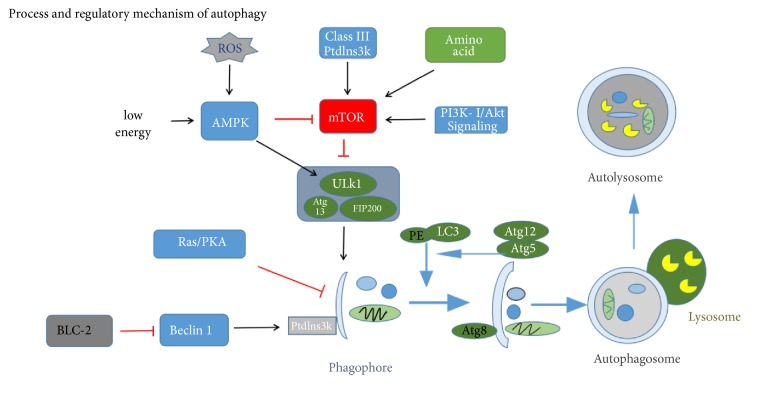
*Process and regulatory mechanism of autophagy*. MTOR inhibits the initiation of autophagy by inhibiting the Atg1 (mammalian ULK1). Various substances and stimuli can induce autophagy through the mTOR pathway, such as amino acids, energy, and oxidative stress. In chondrocytes, when mTOR is activated, it inhibits the expression of ULK1 and then affects the formation of ULK1, FIP200, and Atg13 complexes to inhibit the assembly of autophagic bodies. The function of beclin-1 in autophagy is regulated by Bcl-2, and Bcl-2 inhibits autophagy by combining and isolating beclin-1 under nutrient rich conditions. The induction of autophagy requires the dissociation of beclin-1 from Bcl-2. The formation of phagophore is driven by the beclin-1 associated class III PI3 Kinase with phosphatidylinositol3-phosphate-containing vesicles. The phagophore undergoes elongation and completion driven by two ubiquitin-related conjugation systems, the LC3-PE and Atg12-Atg5. Then, the autophagosome is fused with the lysosome. Finally, the substance degrades in the autolysosome and provides nutrients for the cells.

The autophagy process can be divided into several steps: the induction of autophagy, the formation of autophagic bodies, the fusion of vesicles, and the decomposition of autophagic bodies [4]. Atg protein has a key role in the formation and transport of autophagic bodies. Among the Atg protein, Atg1, Atg6, Atg8 (ULK1, beclin-1, and LC3 in mammals, respectively), and Atg5 are four major regulators of the autophagy pathway. Atg1 kinase domain has an important role in autophagy induction [5]. In mammalian cells, the interaction of Atg13 with ULK1 and ULK2 can mediate the interaction of ULK protein with FIP200. ULK-Atg13-FIP200 complex is the direct target of mTOR and is an important regulator of mTOR signal transduction during autophagy (Figure 1) [6]. By inhibiting the activity of mTOR, the activity of Atg1 kinase is enhanced, and the surrounding related Atg proteins are inserted into the PAS (the site of autophagy formation) to promote the formation of Atg1-Atg13-Atg17 scaffold. The initial assembly of the bilayer membrane requires the III type phosphatidylinositol kinase (PtdIns3K) complex. After vesicle fusion, the degradation of autophagy depends on a series of lysosomal or vacuolar acid hydrolases, including protease A and B. In the case of cell starvation, the small molecules produced by amino acid degradation are transported back to the cytoplasm for protein synthesis and maintenance of cell function.

### 2.1. Regulatory Mechanism of Autophagy

mTOR is an atypical serine/threonine protein kinase involved in phosphoinositide 3-kinase- (PI3K-) related kinase family, which interacts with several proteins to form two different complexes: mTOR complex 1 (mTORC1) and mTOR complex 2 (mTORC2). Previous studies have suggested that mTOR has a negative regulatory effect on autophagy [7]. As mentioned above, mTOR can regulate signal transduction during autophagy through ULK-Atg13-FIP200 complex. Type III phosphatidylinositol enzyme (PI3K) and beclin-1 can be combined to form complexes for autophagosome formation, while BCL-2 and beclin-1 are combined with competitive inhibition of beclin-1 and PI3K to inhibit autophagy activity [8]. Ras/PKA signaling pathway negatively regulates the autophagy activity by regulating the early steps of the Cvt transport process [9]. Furthermore, recent studies have suggested that some nanoparticles may also affect the autophagy in cells [10, 11]. Besides, other relevant articles have provided more detailed description of the processes and mechanisms underlying autophagy [4, 12].

### 2.2. Autophagy Is Involved in Chondrocyte Metabolism

Endochondral ossification is the main form of bone formation in mammals. During this process, chondrocytes undergo a series of differentiation steps to form the growth plate. Briefly, bone marrow mesenchymal stem cells are differentiated into bone progenitor cells and then into chondrocytes that form the cartilage primordia. In turn, they develop into plate-guided bone, which determines the speed and length of bone longitudinal growth. Considering that little blood vessel is present in the growth plate, the cartilage cells are likely to grow in malnourished hypoxic environments [13]. Articular chondrocytes have the function of secreting extracellular mechanism. Among them, the hypertrophic chondrocytes in the growth plate lack the oxygen and energy. During this process, the autophagy activities of cells become active and protect the differentiation of chondrocytes and normal chondrocytes to avoid premature apoptosis and replacement of the bone.

**Figure 2:**
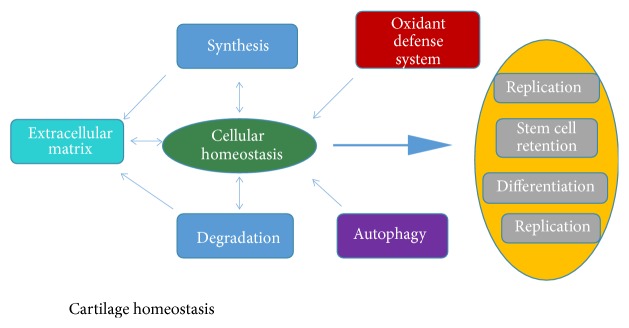
*Chondrocyte homeostasis*. The homeostasis of chondrocytes requires the extracellular matrix, oxidant defense mechanism, and autophagy. There is an inseparable relationship between the synthesis and metabolism of extracellular matrix and the activity of chondrocytes. Chondrocyte homeostasis plays an indispensable role in cell replication, differentiation, and development.

The normal physiological function of the cartilage depends on the healthy chondrocytes. However, the rate of regeneration of chondrocytes is very low after differentiation. Therefore, the homeostasis of cartilage cells is very important for the maintenance of the cartilage tissue function. The survival of chondrocytes depends on the excrete cartilage matrix, so the synthesis and metabolism of cartilage matrix have a certain supportive effect on the homeostasis of cartilage cells [14]. The homeostasis of chondrocytes is essential for the normal replication of the cells, the preservation of the stem cell group, and the normal differentiation (Figure 2).

### 2.3. Autophagy Participates in Energy Metabolism

The levels of intracellular glucose, amino acids and lipids reflect the state of energy and nutrition in the cell and have a key role in regulating metabolism. Autophagy is indispensable for maintaining the metabolic homeostasis of cells (Figure 2) [15]. In order to maintain the material balance in the cartilage cells, substances consumed by autophagy must be supplemented by the synthesis of new macromolecules; and this process is very energy-consuming. In addition, the activation of autophagy is very important for maintaining normal viability of chondrocytes in the state of low energy. In chondrocytes, AMPK (5′-AMP-activated protein kinase) is activated by upstream LKB1 kinase, encoded by the Peutz-Jeghers syndrome gene. An activation of AMPK leads to phosphorylation and activation of TSC1/2 complex in chondrocytes because of the decreased level of intracellular ATP/AMP, which then inhibits mTOR activity by Rheb (Figure 1) [16]. Because of the decreased mTOR activity, autophagy can produce ATP by recycling nutrients from cells and increase the number of ATP in the cells.

In the chondrocytes, the energy is mainly produced by mitochondria. Previous studies have shown that autophagy in mitochondria occurs due to the translocation of E3 ligase Parkin to mitochondria, which activates mitochondrial protein ubiquitination and in turn leads to mitochondrial depolarization, thus affecting the adhesion of P62 and the specificity of mitochondrial autophagy. Moreover, some evidence has suggested that autophagy can effectively regulate the respiration of the mitochondria to achieve the normal ATP level needed by the cell [17], which further indicates that autophagy is essential for the maintenance of cellular energy metabolism. In addition, an experimental study that was performed on human chondrocytes treated with Oligomycin, which is an inhibitor of mitochondrial respiratory chain (MRC) complex V, showed an increase and then a decrease of LC3-II levels (a marker for autophagosome formation) during 24 h and 48 h, respectively, which further suggested a reduced activity of autophagy during mitochondrial dysfunction [18]. Chondrocyte function of autophagy defects causes an accumulation of dysfunctional mitochondria. This indicates that the function of mitochondria and the autophagy activity are interdependent.

The process of producing ATP in normal cell respiration might generate ROS, which can cause loss to mitochondria and lysosomes [19]. Moreover, it is generally believed that besides producing ROS, the mitochondria* are* also involved in the formation of* lipofuscin*. Existing studies have suggested that a decline in autophagy caused by aging may accelerate mitochondrial damage [20]. During aging of cells, lipofuscins are not biodegradable, and the lysosomes rich in lipofuscin consume most of the newly produced lysosomal enzymes. This in turn leads to the decrease of the phagocytosis of the lysosome, lowering the activity of mitophagy. Then, aging mitochondria and inefficient lysosomes accumulate in cells and gradually replace normal organelles, eventually causing cell death due to the lack of ATP. Therefore, this may also be the cause of cartilage damage and apoptosis in elderly patients with OA; however, the entire process needs to be further investigated.

### 2.4. Autophagy Has a Very Important Role in the Synthesis and Metabolism of Protein

When the intracellular amino acids start declining, the amino acids needed in the process of translation are mainly supplied by proteasome degrading proteins. After 6 hours of intracellular amino acid deficiency, amino acids are generated through the lysosomes degradation of proteins [21]. Otherwise, autophagy can affect the synthesis of protein through the mTOR pathway [22, 23]. This suggests that, once the amino acids become absent, cells use the autophagy to obtain new amino acids in order to maintain the normal translations. Not only the autophagy is important for the homeostasis of intracellular amino acids, but also the amino acid itself affects the process of autophagy (Figure 1). For example, in the presence of a large number of amino acids in the human body, MTORC1 is activated and inhibits the induction of autophagy by phosphorylated Atg protein. Glutamine can induce the activation of mTOR by producing *α*-ketoglutarate and can further inhibit autophagy [24]. Moreover, amino acids can also affect autophagy by affecting the expression of autophagy genes [25]. Thus, it appears that, in the process of autophagy by controlling the concentration of amino acids in cells, amino acids can regulate the process of autophagy.

### 2.5. Autophagy in Metabolism of Lipids

Intracellular lipids are stored in lipid droplets (LDs). Autophagy has a major role in controlling the metabolism of lipids, including cholesterol and stearic acid [26]. During nutrient starvation, lipophagy, which is an autophagic process that delivers lipids to the lysosome for their degradation is also of great importance [27–29]. In case the autophagy is inactivated, the lipids accumulate in the cells, which happens because LD participates in the composition of the autophagy pathway [30]. It has been proved that the activation of AHR (aromatics receptor) can induce lipid accumulation [31]. AHR antagonists can reduce the concentration of tissue factor (TF) on the surface of the cell and lead to the inhibition of sterol synthesis [32]. In addition, previous studies have suggested that *β*-adrenergic receptor agonist induces activation of lipophagy by activating RAB7 to initiate lysosomal degradation [33]. Furthermore, it has been shown that, in the state of nutrient insufficiency, RAB7 can direct both autophagosomes and lysosomes to the LD surface to coordinate lipophagy [34]. In addition, itraconazole has been found to interfere with the transport of cholesterol, thus inhibiting the mTOR pathway in autophagy [35].

Otherwise, lipids may also affect the activity of autophagy. The rapid increase of fat in *β*-cells can induce autophagy, while the long-term lipid excess can inhibit autophagy [35]. These results suggest an association between lipid metabolism and the activity of autophagy.

### 2.6. Aging-Related Autophagy in Chondrocytes Metabolism

Aging is one of the major risk factors for OA in adults over 60 years old [36]. The major changes associated with aging in cartilage are similar to those in other tissues, and mainly include impaired chondrocyte biosynthesis and cell death. The gradual accumulation of damaged macromolecules and organelles in somatic cells, which reduces the ability of cells to function and survive, is a core feature of aging-related diseases [15]. With the increase of age, the protection mechanism of the body can also change. The decline of antioxidant defense ability and autophagy damage the metabolic balance of the body and reduce the repair ability of cartilage [37]. Studies have shown that aging causes an autophagy reduction in the chondrocytes. In addition, the autophagy damage induced by aging occurs before the reduction of chondrocytes and structural damage [37–39]. In chondrocytes, mTOR inhibition by rapamycin protect from the oxidative stress and cell death [18, 40, 41]. A recent study has found that REDD1 deficiency reduces the autophagy protein expression, regulates mitochondrial biogenesis in chondrocytes, and increases the severity of experimental osteoarthritis [42].

Although the major role of autophagy in chondrocytes is well understood, the exact molecular mechanisms of action still remain unclear. The newly emerged metabonomics allows for the use of NMR spectroscopy and mass spectrometry to qualitatively and quantitatively analyze the important metabolites in autophagy process [43]. We believe that future studies would benefit from the metabolomics to study the mechanism of autophagy in regulating the metabolites in chondrocytes. This would provide a new approach to study of the autophagy in chondrocytes metabolism.

### 2.7. The Effect of Autophagy on Osteoarthritis

Chondrocytes are the sole cellular constituents of normal cartilage in mammals. Chondrocytes are not easy to regenerate after they are differentiated, and their damage has been associated with OA [44]. The main reasons for the occurrence of OA are mechanical wear of human joints and age related cartilage degeneration [45]. Studies have suggested that the decrease in autophagy increases the risk of cartilage degeneration in diabetic patients [46]. In addition, decreased expression of ULK1, beclin-1, and LC3 has been observed between normal chondrocytes and chondrocytes with OA [38]. Also, different activity of autophagy has been found in different regions of OA cartilage [47]. The low level of autophagy in OA patients has been associated with apoptosis and mitochondrial changes [48]. More importantly, during cartilage degeneration, the level of gene expression associated with autophagy is reduced. This also confirms that the activity of autophagy in the cartilage cells declines with aging [49]. The mechanism of aging leading to the decline of autophagy is mainly related to the failure of hydrolases in lysozyme, which results in the increase of toxic protein products and the elimination of autophagosomes in aging tissues [50]. Nevertheless, the main function of autophagy is to transform damaged organelles and proteins into substances that cells need, so as to protect the organism from normal and pathological senescence. Additionally, it has been found that autophagy may have both a cytoprotective and death-promoting role in chondrocytes [51]. MSU crystals can cause chondrocyte death through the activation of autophagy process [52]. Overactivated autophagy can lead to cell death [53]; however, there have been no specific experiments to prove this in chondrocytes.

As demonstrated by previously published reports, the signal transduction pathway of PI3K/AKT/mTOR has shown to be involved in the mechanism of chondrocyte death regulation in the OA rat model [54, 55]. In addition, the expression of beclin-1 inhibits the senescence of cartilage cells by inhibiting the PI3K/Akt/mTOR signaling pathway to promote cell viability [56]. The long-term use of dexamethasone may induce degeneration of cartilage and induce autophagy as a defense mechanism of chondrocytes in response to dexamethasone induced ROS [57]. Therefore, enhancing autophagy to maintain homeostasis and homeostasis in chondrocytes is a feasible way to prevent osteoarthritis from aging-related cell dysfunction.

At present, some drugs have shown the ability to delay the aging and damage of chondrocytes by enhancing autophagy. For example, rapamycin can inhibit mTORC1 and activate the autophagy. In the mouse OA model, rapamycin can regulate the signal transduction of mTOR during autophagy, thereby reducing the degree of damage to the cartilage. In addition to the protection of articular cartilage, rapamycin can also reduce the severity of knee synovitis in mice [58]. Yet, the long-term use of rapamycin may induce other diseases, which may be related to its ability to inhibit the immune function [59]. Recent studies have found that local injection of rapamycin in the joint can delay the degeneration of the joints in mice, which may be a potential way to prevent OA [60]. In addition, glucosamine can enhance autophagy by inhibiting the mTOR pathway, and it has an anti-inflammatory effect and promotes the metabolism of cartilage cells [61]. The long-term use of glucosamine is not harmful to people with impaired glucose tolerance [62]. Recently, Cheng et al. [63] have found that Torin 1 enhances the expression level of beclin-1 and LC3, and autophagy was more obvious. The increase of chondrocyte autophagosomes inhibits the degenerative changes of knee joint and may serve as an effective therapeutic drug for OA.

### 2.8. Conclusion

Autophagy is critical for material metabolism of chondrocytes. Autophagy can repair the damage of chondrocytes and inhibit their aging and has an antagonistic effect on the occurrence and development of OA. Although great progress has been made in understanding osteoarthritis, the current understanding of autophagy and chondrocyte metabolism is still at an early stage. Based on the rapid development of metabonomics, it is possible to use nuclear magnetic resonance spectroscopy and mass spectrometry to study the relationship between autophagy and material metabolism and to discuss the prevention and treatment of OA by the effect of autophagy on cell metabolism.
